# *Pratylenchus Araucensis* (Rhabditida: Pratylenchidae) a Widely Distributed Nematode in *Musa* Spp. from Colombia

**DOI:** 10.2478/jofnem-2022-0057

**Published:** 2023-01-08

**Authors:** C. E. Arboleda-Riascos, D. H. Riascos-Ortiz, F. Varón De Agudelo, A. T. Mosquera-Espinosa, C. M. G. Oliveira, J. E. Muñoz-Flórez

**Affiliations:** 1 Universidad Nacional de Colombia, Palmira, Colombia; 2 Universidad del Pacífico, Buenaventura, Colombia; 3 Corporación Colombiana de Investigación Agropecuaria-AGROSAVIA, Pasto, Colombia; 4 Departmento de Ciencias Naturales y Matemáticas, Pontificia Universidad Javeriana, Cali, Colombia; 5 Laboratorio de Nematologia, Instituto Biológico, Campinas, São Paulo, Brazil

**Keywords:** *COI* mtDNA, *D2*–*D3* expansion segment of LSU rDNA, molecular biology, morphology, plantain

## Abstract

*Pratylenchus* is one of the most limiting nematodes of Musaceae production in the world. Knowledge of the nematode species is one of the requirements for its management in the field. This study aimed to identify up to the species level *Pratylenchus* populations associated with plantain and banana crops in the states of Caldas, Quindío, and Valle del Cauca in Colombia. In these regions, *Pratylenchus* has been reported to affect these crops in the past, but with records of the nematode only up to the genus level. For this purpose, five populations of *Pratylenchus* extracted from samples composed of roots and rhizospheric soil, four from plantain and one from banana, were identified through morphological, morphometric, and molecular analysis (sequencing of the *D2*–*D3* of rRNA and cytochrome oxidase subunit I of mtDNA). All populations were identified as *P. araucensis*, a species reported previously in eastern Colombia, and one that the present study found in the center and southwest of the country, indicating that this species of nematode is widely distributed in the Musaceae-producing areas of Colombia. The present study reports the first *COI* mtDNA sequences for this species of nematode.

*Pratylenchus* is a migratory endoparasitic nematode that limits the production of a wide range of crops in tropical and subtropical areas of the world (Sasser and Freckman, 1987; Castillo and Vovlas, 2007; Múnera *et al*., 2009). Crops affected by *Pratylenchus* include different species of the Musaceae family, including plantain (*Musa paradisiaca* [L.] AAB Simmonds cv. Dominico Hartón) and banana (*Musa acuminata*), in which the nematode damages the root system of the plant (Riascos-Ortiz *et al*., 2022). Symptoms induced by the nematode in Musaceae roots include initial internal yellow lesions that later turn purple and finally brown. Externally, the necrotic areas of the roots appear black due to the destruction of the cortical tissues (CABI, 2022).

In affected plantain and banana plants, the nematode reduces the root system and the ability to take up water and nutrients, which causes foliar chlorosis, growth retardation, decreased bunch weight, lengthening of the productive cycle, and returns or weak suckers (Múnera, 2008; De Luca *et al*., 2012). In fields severely infested with the nematode, plants suffer toppling and complete bunch loss (CABI, 2022). Production losses are approximately 15%, although they can be higher in plantations >3-yr old and with little agronomic management (Guzmán and Castaño, 2004).

In addition to the direct damage caused to plantain and banana roots, a high correlation has been recorded between *Pratylenchus* and infection by fungi such as *Fusarium oxysporum*, *F. redolens, F. sambucium, Nigrospora musae*, and *Rhizoctonia solani*, and also bacteria such as *Xanthomonas campestris* (Bridge *et al*., 1997).

Four *Pratylenchus* species have been reported to affect plantain and banana crops in the world: *P. araucensis* Múnera, Bert & Decraemer, *P. speijeri* De Luca *et al*.*, P. coffeae* Zimmermann, and *P. goodeyi* Sher and Allen (Zimmermann, 1898; Moens *et al*., 2006; Souza, 2008; Múnera, 2008; De Luca *et al*., 2012; Luambano *et al*., 2019; Handoo *et al*., 2021). Of these species, only two have been reported in Colombia, *P. coffeae* (in Santa Marta, Urabá, coffee zone [Quindío] and Caquetá) and *P. araucensis* (in Arauca), with morphometric and molecular support only for the latter species (Barriga and Cubillos, 1980; Múnera *et al*., 2009).

The identification of these species is not an easy task due to their morphological and morphometric similarities (overlap of characteristic between species). This condition has led some of these species to be considered cryptic, including *P. speijeri* and *P. coffeae*, which are morphologically indistinguishable; and a clear separation is possible only through molecular analysis (De Luca *et al*., 2012). Other species can be separated using a few characteristics of diagnostic value; examples of this mode of distinguishing would be the case of *P. araucensis* that differs from *P. speijeri* by the length of the stylet (14.7– 15.9 μm vs. 16.5–18.0 μm), from *P. coffeae* by the position of the vulva (78% vs. 80%), and from sister species such as *P. jaehni* and *P. loosi* mainly by body length (462 μm vs. 488 μm and 522 μm) (Múnera *et al*., 2009; De Luca *et al*., 2012).

*Pratylenchus araucensis* was identified by integrative taxonomy attacking Musaceae in the eastern region of Colombia (Múnera *et al*., 2009). Although other works have registered *Pratylenchus* affecting Musaceae in the country, these reports lack morphometric and molecular information, restricting the report of the nematode to the genus level (Zuñiga *et al*., 1979; Guzmán and Castaño, 2004, 2007; Riascos-Oritiz *et al*., 2021). This indicates that there is a lack of knowledge of the species of *Pratylenchus* associated with Musaceae in Colombia, which could make it difficult to manage nematode populations in the main fruit-production areas in the country.

Therefore, it is necessary to expand at the species level the taxonomic knowledge of *Pratylenchus* populations associated with plantain and banana in Colombia, especially in the fruit-production areas of the center and southwest, where reports of the nematode have been developed up to the genus level. Against this background, the present study raised the following objectives: (i) to identify populations of *Pratylenchus* from the central and southwestern areas of Colombia through morphological, morphometric, and molecular analysis; (ii) to ascertain the intraspecific diversity of *Pratylenchus* species identified from molecular data; and (iii) to ascertain the evolutionary relationships of the analyzed populations based on the *D2*–*D3* segment of LSU ribosomal DNA (rDNA) and *COI* mtDNA.

## Materials and Methods

### Sampling and morphological and morphometric identification

Composite samples of rhizosphere soil and roots were collected from plantain and banana crops located in the states of Valle del Cauca (municipality of Buenaventura [85 m.a.s.l., average temperature of 26°C, and annual rainfall of 9,000 mm]), Caldas (municipality of Palestina [1,050 m.a.s.l., average temperature of 18°C, and annual rainfall of 2,859 mm]), and Quindío (municipality of Calarcá [1,573 m.a.s.l., average temperature of 19°C, and annual rainfall of 2,500 mm]), Colombia, during the year 2018. Each sample of approximately 1 kg was composed of subsamples extracted from 15 to 20 plants per hectare, which were randomly selected. The samples were taken at 25 cm from the pseudostem and at a depth between 0 cm and 30 cm at three equidistant points.

The extraction of the phytonematodes was carried out using the modified Cobb method (Varón de Agudelo and Castillo, 2001; Ravichandra, 2014). Subsequently, the nematodes were killed by exposure to 65°C for 4 min and fixed in 2% formalin (Rosa *et al*., 2014; Riascos-Ortiz *et al*., 2019). For each population, morphometric data were recorded for different diagnostic characteristic (Castillo and Vovlas, 2007; Múnera *et al*., 2009).

## Statistical analysis

The morphometric data recorded in this study and others taken from the literature (Inserra *et al*., 2001; Múnera, 2008; De Luca *et al*., 2012) were statistically analyzed using principal component analysis (PCA) to establish possible groupings and discriminant diagnostic characteristic that allow identifying the populations studied at the species level, using version 9.4 of the developed by the SAS institute statistical package.

### Molecular analysis

For the DNA extraction, the protocol proposed by Riascos-Oritiz *et al*. (2019) was used. For this purpose, a single specimen was cut and transferred to a tube with 15 μl of lysis buffer (50 Mm KCl, 10 Mm Tris pH 8.3; 2.5 Mm MgCl_2_; 0.45% NP 40; 0.45% Tween 20; 60 μg/ml proteinase K). Subsequently, the tube was incubated at -80°C (15 min), 65ºC (1 hr), and 95°C (15 min). After this, the tube was centrifuged (1 min at 16,000 g) and stored at -20°C. Using PCR, the *D2*–*D3* region of the large subunit (LSU) of rDNA (28S) was amplified using forward primer D2A (5´-ACAAGTACCGTGAGGGAAAGTTG-3´) and reverse primer D3B (5´-TCCTCGGAAGGAACCAGCTACTA-3´) (De Ley *et al*., 1999). The cytochrome oxidase subunit I region of mitochondrial DNA (*COI*) was amplified using forward primer JB3 (5´-TTTTTTGGGCATCCTGAGGTTTAT-3´) and reverse primer JB4.5 (5´-TAAAGAAAGAACATAATGAAAATG-3´) (Bowles *et al*., 1992). The PCR conditions were initial denaturation during 2 min at 94°C followed by 40 cycles of 45 sec at 94°C, 45 sec at 55°C, 1 min at 72°C, and final extension of 10 min at 72°C for the *D2*–*D3* region; and initial denaturation during 2 min at 94°C followed by 40 cycles of 45 sec at 94°C, 45 sec at 54°C, 1 min at 72°C, and final extension of 10 min at 72°C for *COI*. The PCR products were sequenced in both directions by Bioneer Corporation, Daejeon, South Korea.

## Phylogenetic analysis

The consensus sequences were edited using the BioEdit 7.0.5.3 (Hall, 1999). Once the sequences were refined, their identity was confirmed by comparing them with the GenBank database, using the BLAST software (http://www.ncbi.nlm.nih.gov/BLAST). Subsequently, the sequences presented under the accession numbers in Table 1 were manually aligned using MEGA6 (Tamura *et al*., 2013). Based on the matrix obtained for the two genes used, it was possible to determine the nucleotide substitution model, taking into account the Bayesian information criterion (BIC) using the Model Generator v.0.851 software (Keane *et al*., 2006). The phylogenetic analysis was determined using the maximum likelihood (ML) method together with the Kimura 2-parameter model, and the internal reliability of the nodes was determined by using the method with 1,000 interactions. As an external group of the phylogenetic tree of the LSU partial region, the species *Belonolaimus longicaudatus* (KF963100) was used and for *COI* the species *Mesocriconema xenoplax* (MG422913).

**Table 1:** Morphometric data of studied and reference populations of *Pratylenchus araucensis*.

Characteristic	Plantain, Zabaletas - Buenaventura	Plantain, Delfina - Buenaventura	Plantain, Caldas	Plantain, Calarcá - Quindío	Banana, Calarcá - Quíndio	*Musa* sp. Arauca, type population (Múnera *et al.*, 2009)
	*n* = 19	*n* = 14	*n* = 19	*n* = 15	*n* = 10	*n* = 40
L	456 ± 24.0	501.8 ±56.0	484.5 ± 24.7	549.2 ± 13.9	551.3 ± 13.5	462 ± 31
	(401.2 - 496.3)	(449.3-668.7)	(444.4 - 538.0)	(518.4-578.3)	(534.7 - 570.4)	(376-511)
a	26.3 ±2.1	28.2 ± 1.9	29.9 ±2.0	25.2 ±1.3	24.8 ± 1.4	23.9 ±2.3
	(20 - 29.8)	(23.5-31)	(23.0-32.8)	(21.6-26.9)	(21.6-26.3)	(19.6-29.2)
b	5.8 ±1.0	6.1 ±0.7	6.4 ±0.5	6.2 ±1.7	6.7 ±1.4	6.2 ±0.6
	(4.7-7.0)	(5.1 -6.9)	(4.9 - 7.3)	(5.1 -7.4)	(5.5-7.7)	(4.8 - 7.8)
b'	4.4 ±0.6	4.7 ±0.2	4.3 ±0.5	4.1 ±0.8	4.5 ±1.0	4.7 ±0.4
	(4.0-5.7)	(3.7-6.0)	(3.7-5.2)	(3.7-5.0)	(4.0 - 4.9)	(3.6 - 5.9)
c	17.3 ±1.2	18.2 ± 1.5	18.9± 1.3	18.3 ±1.0	17.5 ±0.4	17.6 ±1.4
	(14.1 -22.3)	(14.9-21.8)	(15.2-23.4)	(16.7-20.2)	(16.6-18.0)	(14.5-21.1)
c'	2.3 ±0.5	2.5 ±0.4	2.6 ± .02	2.1 ±0.1	2.2 ±0.1	2.3 ±0.3
	(1.4-2.8)	(1.7-3.0)	(1.9-3.1)	(2.0-2.3)	(2.1 -2.5)	(1.7-2.9)
V%	76.7 ±1.8	77.6 ± 1.1	80.1 ± 1.6	76.5 ± 1.95	74.7 ±0.6	78 ± 1.2
	(73.9-80.1)	(75.7 - 79.4)	(76.5 - 83.6)	(72.9-81.4)	(74.0 - 75.9)	(75 - 80)
Stylet length	12.1 ±0.6	12.6± 1.1	11.9 ±0.6	14.4 ±0.4	14.7 ±0.4	15.3 ±0.4
	(11.1 -13.1)	(10.7-14.0)	(10.6-12.9)	(13.5-14.9)	(14.2-15.6)	(14.7-15.9)
Tail length	26.3 ±2.0	27.5 ± 1.8	25.7 ± 1.8	30.1 ±2.0	31.5 ±0.8	26.3 ±2.5
	(20.9-29.6)	(24.0-31.1)	(22.0-29.4)	(27.1 -34.6)	(30.2-32.7)	(21.5-31.9)
DGO	2.8 ±0.6	2.9 ±0.5	2.6 ±0.4	2.3 ±0.1	2.5 ±0.1	2.7 ±0.4
	(2.3-4.1)	(2.1 -3.6)	(2.0-3.2)	(2.1 -2.5)	(2.3-2.6)	(1.8-3.1)
Maximum body	16.7 ±1.5	17.8 ±2.0	15.7 ± 1.2	21.8 ±1.4	22.3 ± 1.3	17.0 ±1.8
diameter	(13.7-19.7)	(14.0-20.5)	(12.3-17.2)	(20.5-26.0)	(21.2-24.7)	(13.5-20.9)
Anal body	11.1 ±0.8	11.6 ±0.9	10.2 ± 0.8	14.1 ±0.6	14.1 ± 0.6	11.4 ± 1.5
diameter	(9.7-12.5)	(10.0-13.2)	(9.0 - 11.5)	(13.1 -15.2)	(13.2-14.8)	(8.6-17.2)
Number of head	2.0 ±0.0	2.0 ±0.0	2.0 ±0.0	2.0 ±0.0	2.0 ±0.0	
annuli	(2.0-2.0)	(2.0-2.0)	(2.0-2.0)	(2.0-2.0)	(2.0-2.0)	
Lip region width	6.1 ±0.5	7.1 ±0.9	6.3 ±0.5	8.2 ±0.4	8.1 ±0.3	7.2 ±0.3
	(4.7-6.8)	(5.6-9.1)	(5.5 - 7.3)	(7.7-8.9)	(7.5-8.5)	(6.7 - 7.4)
Lip region height	2.5 ±0.6	2.8 ±0.3	2.5 ±0.4	2.1 ±0.2	2.4 ±0.2	2.5 ±0.7
	(1.5-3.6)	(2.3-3.2)	(1.8-3.1)	(1.8-2.4)	(1.9-2.6)	(2.0-4.0)
Pharyngeal gland	35.7 ±5.3	31.7 ± 1.4	32.8 ±4.7	28.6 ±3.6	40.5 ± 1.1	24.2 ± 7.7
length	(25.8-45.7)	(30.1 -34.4)	(24.0-39.2)	(24.1 -33.8)	(38.9-42.2)	(10.4-46.6)
PUS	18.5 ±5.5	31.2 ± 1.6	24.5 ±4.8	29.6 ±0.8	30.4 ± 1.2	18.1 ±4.0
	(8.4-27.3)	(29.8-33.2)	(14.8-32.0)	(28.6 - 31.4)	(28.8-32.0)	(9.8-24.5)

Measurements in micrometers; mean ± s.d. (range).

## Results

### Morphological and morphometric identification

Five populations of *Pratylenchus*, four from plantain (two from Valle del Cauca, one from Caldas, and one from Quindío) and one from banana (from Quindío), were identified as *P. araucensis*. The morphological and morphometric data from the analyzed populations were similar to the data reported in the original description of *P. araucensis* (Table 1).

**Figure 1:**
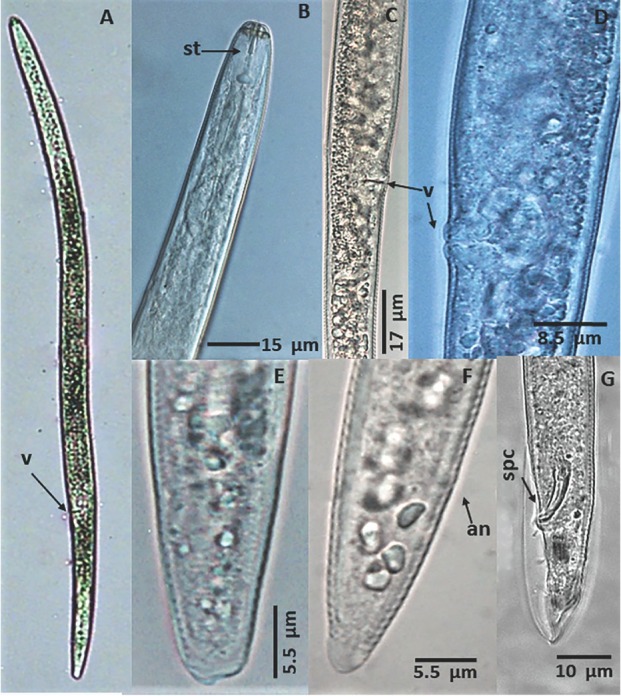
*Pratylenchus araucensis*. **(A–F):** Female. **(A)**. Body of a female, **(B)**. Anterior body region, **(C** and **D)**. Vulval region. **(E** and **F)**. Posterior region. **(G)**. Posterior region of the body of a male. St = Stylet, v = Vulva, an = Anus, spc = Spicule.

The populations identified in the present study as *P. araucensis* were morphologically characterized by the presence of a body that was slender and vermiform (Fig. 1A), flattened labial region with presence of two annuli, basal knobs of the stylet rounded to oblong (Figs. 1B,C), vulva located posteriorly (Figs. 1D–F), round spermatheca, the shape of the tail conoid to subcylindrical, end of the tail rounded to truncated (Figs. 1G–J), and presence of males (Fig. 1G).

**Figure 2:**
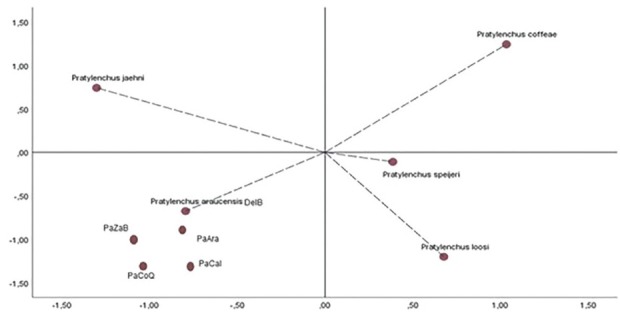
Biplot (separation) based on morphometric data of *Pratylenchus araucensis* populations. Populations of *P. araucensis* from Caldas, Quindío, and Valle del Cauca were assigned to their corresponding species based on morphometric data. The first two PCA axes are shown. Pa = *Pratylenchus araucensis*, Ara = Arauca, Cal = Caldas, CoQ = Quindío, ZaB = Zabaletas (Valle del Cauca [Buenaventura]), and DelB = Delfina (Valle del Cauca [Buenaventura]). PCA, principal component analysis.

The multivariate statistical analysis showed that the populations identified as *P. araucensis*, from the states of Valle del Cauca, Caldas, and Quindío, had such morphological characteristics to be grouped in the same cluster with the type population of the same species previously recorded in the department of Arauca, Colombia, and to be grouped apart from other species reported in Musaceae (Fig. 2), such as *P. coffeae* and *P. speijeri* (Souza, 2008; Múnera *et al*., 2009; De Luca *et al*., 2012).

According to PCA, the main components 1, 2, and 3 presented eigenvalues ≥1, explaining 87% of the variation. Thus, the characteristic with diagnostic values to delimit between the species *P. araucensis*, *P. coffeae*, *P. jaehni*, and *P. loosi* are: post-uterine sac length, total length, tail length, maximum width of the body, and width of the body at the level of the anus in the main component (CP)1; stylet length, vulva position, and index c at CP2; and width of the cephalic region and index a in CP3 (Table 2). These variables, according to the results of the multivariate analysis, are characteristic of diagnostic value that allow delimiting between the analyzed species.

**Table 2:** Correlations between the first three main components and the morphometric parameters of *P. araucensis* females.

Variable	Main component		
	Prin1	Prin2	Prin3
Stylet length (μm)	0.298	**0.440**	-0.159
Vulvar position (%)	-0.288	**0.431**	0.178
Post-uterine sac length (μm)	**0.315**	0.176	0.407
Overall length	**0.330**	0.370	0.245
Tail length (μm)	**0.379**	-0.046	0.235
Maximum body width (μm)	**0.396**	0.005	-0.182
Width body anus (μm)	**0.377**	0.133	-0.188
Head region width (μm)	0.242	-0.345	**0.447**
Index a (μm)	-0.294	0.122	**0.620**
Index c (μm)	-0.182	**0.547**	-0.097

Numbers in bold format correspond to discriminant characteristic.

## Molecular identification

A total of 33 DNA sequences was obtained in the present study, 16 corresponding to the *D2*–*D3* segment of rDNA and 17 for *COI* of mitochondrial DNA (Table 3). All the sequences of the *D2*–*D3* segment showed a similarity of 99.73% with reference sequences of *P. araucensis* (FJ463261, FJ463260, and FJ463258). The taxonomic identity of the analyzed populations could not be resolved based on sequences corresponding to *COI*, because the present study reports the first *COI* sequences for the species *P. araucensis*.

**Table 3:** Information of D2–D3 rDNA and COI mtDNA regions downloaded from GenBank and obtained in the present study.

Species name	Location	D2–D3 (LSU) accession number	COI accession number	Reference or source
*Pratylenchus araucensis*	Colombia	MZ636668; MZ636669 MZ636670; MZ636671 MZ636672; MZ636673 MZ636674; MZ636675 MZ636676; MZ636677 MZ636678; MZ636679 MZ636680; MZ636681 MZ636682; MZ636683	MZ636684; MZ636685 MZ636686; MZ636687 MZ636688; MZ636689 MZ636690; MZ636691 MZ636692; MZ636693 MZ636694; MZ636695 MZ636696; MZ636697 MZ636698; MZ636699 MZ636700	Present study
*P. araucensis*	Colombia	FJ463262; FJ463261; FJ463260; FJ463275	-	Múnera *et al*. (2009)
*P. hippeastri*	China	KR029084	KY424099	Wang *et al*. (2016)
*P. hippeastri*	China	KP161608	KY424098	Unpublished
*P. hippeastri*	South Africa	MH324472	-	Shokoohi (2018)
*P. hippeastri*	Japan	KC796706; KC796707	-	Wujian *et al*. (2014)
*P. scribneri*	USA	KX842632	-	Huang and Yan (2017)
*P. scribneri*	China	MK209593	-	Li *et al*. (2018)
*P. scribneri*	China	-	KY424093	Unpublished
*P. scribneri*	China	-	KX349425	Liu *et al*. (2016)
*P. pseudocoffeae*	Korea	KT175532; KT175533	-	Kim *et al*. (2016)
*P. pseudocoffeae*	Costa Rica	KT971360	-	Zamora et at. (2016)
*P. pseudocoffeae*	China	-	KY424089	Unpublished
*P. loosi*	China	KF430796; KF430797	-	Unpublished
*P. loosi*	Italy	-	LR215647; LR215648	Unpublished
*P. coffeae*	China	MN592778; MN227243; MN588282		Li *et al*. (2020)
*P. coffeae*	China	-	KX349421	Liu *et al*. (2016)
*P. coffeae*	China	-	MN366418	Unpublished
*P. coffeae*	USA	-	KU198943; KU198942	Troccoli *et al*. (2016)
*P. speijeri*	China	KF974701; KF974698; KF974697		Unpublished
*P. speijeri*	China		KY424088; KY424087	Unpublished
*P. penetrans*	USA	KY969632; KY969631		Baidoo *et al*. (2017)
*P. penetrans*	Korea	-	MN746802; MN746803; MN746801	Mwamula *et al*. (2020)
*P. kumamotoensis*	Korea	KT175529; KT175530; KT175525	-	Kim *et al*. (2016)
*P. parazeae*	China	KP903444; KP903445; KP903443; KP903442		Wang *et al*. (2015)
*P. parazeae*	China		KY424116; KX349424; KY424115	Unpublished
*P. zeae*	Brazil	MW363009; MW363001	-	Abade (2020)
*P. zeae*	China		KX349414; KX349416; KX349419	Liu *et al*. (2016)
*P. horti*	Belgium	-	MK114154; MK114153; MK114152	Nguyen *et al*. (2018)
*P. brachyurus*	China	-	KX349420; KY424082; KY424083; KY424080	Unpublished

LSU, large subunit; rDNA, ribosomal DNA.

Based on the analysis of 16 partial sequences of the 746 bp *D2*–*D3* segment, from three geographic regions, Valle del Cauca, Caldas, and Quindío, an intraspecific variation for *P. araucensis* of 0.27% (2 bp) was observed between populations of Valle del Cauca (Buenaventura) concerning the populations of Caldas, Quindío, and Arauca. Similarly, based on 17 partial sequences of the *COI* gene, from the same geographical areas, with a size of 393 bp, greater intraspecific variability was determined, with a value of 2.04% (8 bp) between the populations of Valle de Cauca (Buenaventura) and those of Caldas and Quindío (coffee region).

## Phylogenetic analysis

**Figure 3:**
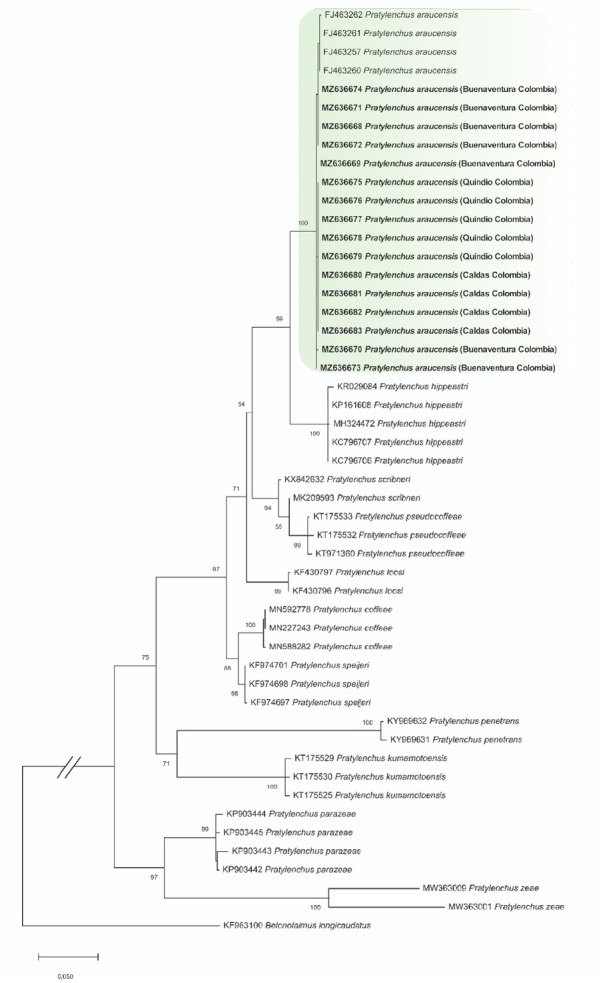
Phylogenetic tree obtained by maximum likelihood of the *D2*–*D3* (LSU) partial region of species of the genus *Pratylenchus*. The isolates corresponding to this work are marked in bold. The numbers above nodes indicate bootstrap values >70%. LSU, large subunit.

**Figure 4:**
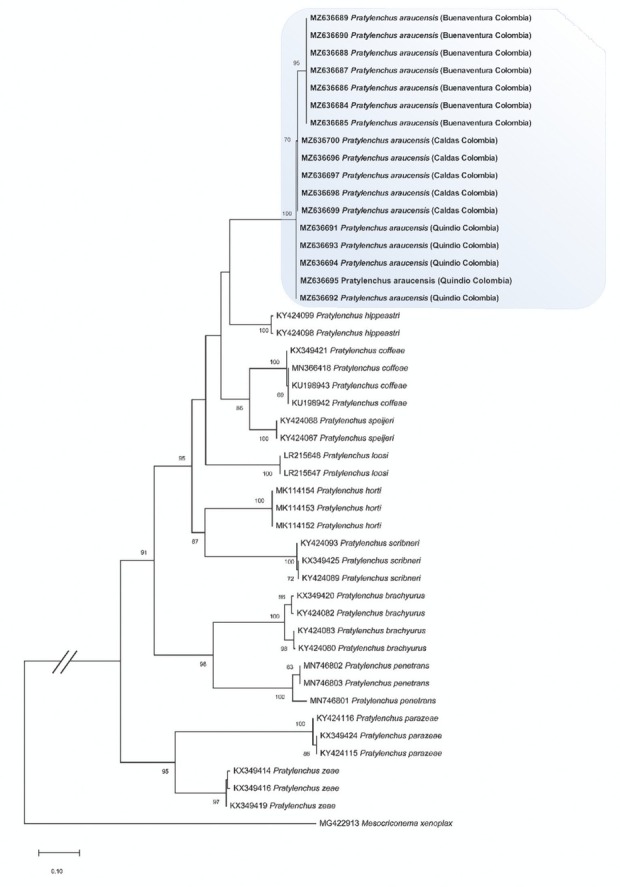
Phylogenetic tree obtained by maximum likelihood of the *COI* partial region of species of the genus *Pratylenchus*. The isolates corresponding to this work are marked in bold. The numbers above nodes indicate bootstrap values >70%.

The alignment based on the LSU region comprised a total of 50 taxa with 837 characters analyzed. For the *COI* region, it included 47 taxa, with a total of 470 characters. The phylogenetic analysis based on the *D2*–*D3* segment allowed the grouping of the sequences obtained in the present study with the species *P. araucensis* with bootstrap support on the branch of 100% and apart from other morphologically similar species such as *P. coffeae*, *P. speijeri* and *P. loosi* (Fig. 3). The populations based on the phylogeny constructed for the *COI* gene were grouped in a clade apart from other *Pratylenchus* species and internally separated by geographic origin (Fig. 4).

## Discussion

Species of *Pratylenchus* are difficult to separate from each other, due to their similarity in morphometrics and morphology, although the genus is easily recognizable (Castillo and Vovlas, 2007). However, based on an integrative taxonomy approach, including morphological, morphometric, and molecular characterization, the populations obtained in this study associated with *Musa* spp. crops were identified as *P. araucensis*. The morphological characteristics and morphometric data recorded for the analyzed populations are similar to those reported for the type population (Múnera *et al*., 2009).

The results indicated that *P. araucensis*, reported previously for the first time in the state of Arauca (eastern region of Colombia), is also present in the states of Quindío, Caldas (central region of Colombia), and Valle del Cauca (southwestern region of Colombia). The foregoing may be related to the movement of corms infested with the nematode between producing areas given the form of clonal propagation of plantain and banana crops (Riascos-Ortiz *et al*., 2022).

The presence of *P. araucensis* in plantain-producing areas with contrasting climatic conditions indicates that this species has a wide range of adaptation to temperature (from 18°C in Caldas and Quindío to 26°C in Valle del Cauca), rainfall (as confirmed by pluviometric measurements ranging from 2,500 mm/yr in Caldas and Quindío to 9,000 mm/yr in Valle del Cauca), and altitude (from 7 m.a.s.l in Valle del Cauca to 1,573 m.a.s.l in Caldas and Quindío) (Orión *et al*., 1979, 1984; Glazer and Orion, 1983; Talavera *et al*., 1998; Orión, 2000; Múnera *et al*., 2009; Bucki *et al*., 2020).

The populations analyzed in this research were identified through the use of morphological and morphometric characteristic. PCA separated the species identified in this study from others that are morphologically and morphometrically similar, showing the importance of morphological and morphometric data, in addition to multivariate statistical analysis for the identification of species of the genus *Pratylenchus* (Castillo and Vovlas, 2007; Múnera *et al*., 2009; Singh *et al*., 2018; Qing *et al*., 2019; Riascos-Oritiz *et al*., 2019).

The analysis of the main components indicated that among the diagnostic characteristic with the highest resolution to discriminate or delimit between some species reported in Musacaes are the length of the post-uterine sac, total length of the body, length of the tail, maximum width of the body, width of the body at the level of the anus, length of the stylet, position of the vulva, index c, width of the cephalic region, and index a, mainly. These variables, except the width of the cephalic region, are considered of diagnostic value in the separation of *P. araucensis* from *P. coffeae*, *P. jaehni*, and *P. loosi* (Múnera *et al*., 2009).

Based on the sequence of the *D2*–*D3* segment of rRNA, it was confirmed that the populations analyzed belong to the species *P. araucensis*. According to the phylogenies constructed for both genes, and using molecular data generated in this study and others obtained from GenBank, *P. araucensis* is separated from different sister species of the same genus, including some reported in Musaceae. These results are consistent with those recorded for the morphological and morphometric analysis (Múnera *et al*., 2009). The phylogenetic tree generated by *COI* molecular data showed that the populations of *P. araucensis* analyzed in this research were grouped by geographic origin, indicating the existence of intraspecific diversity in this species.

Different research works have established the levels of intraspecific diversity for various species of *Pratylenchus*. For example, Kolombia *et al*. (2020) reported a low intraspecific variability of 0 bp to 8 bp (0%–1.93%) among populations of *P. hexincisus* from China, Italy, and the United States and a high intraspecific variability of 54 bp to 102 bp (19.1%–24.5%) among populations from the same countries. Mirghasemi *et al*. (2019) reported a low intraspecific variability of 0.0% to 0.96% (0–4 bp) between populations of *P. loosi* from Iran, China, and Japan. Based on the results of these investigations, the intraspecific variability recorded for *P. araucensis* in the present study can be considered low. However, the nucleotide differences between populations of *P. araucensis* from Valle del Cauca and those from Caldas and Quindío may be related to contrasting climatic differences between regions, specifically rainfall (Castillo and Vovlas, 2007; Mirghasemi *et al*., 2019).

## References

[j_jofnem-2022-0057_ref_001] Abade C. L. P. (2020). Caracterização morfométrica e molecular de Pratylenchus zeae em cana-de-açúcar na região nordeste e controle biológico de nematoides em milho. Tese Doutorado.

[j_jofnem-2022-0057_ref_002] Baidoo R., Yan G., Nagachandrabose S., Skantar A. M. (2017). Developing a real-time PCR assay for direct identification and quantification of Pratylenchus penetrans in soil. Plant Disease.

[j_jofnem-2022-0057_ref_003] Barriga R., Cubillos G. (1980). Principales nematodos fitoparásitos asociados con cultivos de plátano (Musa AAB y Musa ABB) en cuatro regiones de Colombia. Fitopatologia Colombiana.

[j_jofnem-2022-0057_ref_004] Bowles J., Blair D., McManus D. P. (1992). Genetic variants within the genus Echinococcus identified by mitochondrial DNA sequencing. Molecular and Biochemical Parasitology.

[j_jofnem-2022-0057_ref_005] Bridge J., Fogain R., Speijer P. (1997). Nematodos lesionadores de los bananos. Plagas de Musa. Hoja divulgativa No. 2.

[j_jofnem-2022-0057_ref_006] Bucki P., Qing X., Castillo P., Gamliel A., Dobrinin S., Alon T., Braun S. (2020). The genus Pratylenchus (Nematoda: Pratylenchidae) in Israel: From taxonomy to control practices. Plants.

[j_jofnem-2022-0057_ref_007] (2022). Pratylenchus coffeae (banana root nematode). Invasive species compendium. Datasheet.

[j_jofnem-2022-0057_ref_008] Castillo P., Vovlas N. (2007). Pratylenchus (Nematoda: Pratylenchidae): Diagnosis, biology, pathogenicity and management (Nematology monographs and perspectives).

[j_jofnem-2022-0057_ref_009] De Ley P., Felix M. A., Frisse L. M., Nadler S. A., Sternberg P. W., Thomas W. K. (1999). Molecular and morphological characterisation of two reproductively isolated species with mirror-image anatomy (Nematoda: Cephalobidae). Nematology.

[j_jofnem-2022-0057_ref_010] De Luca F., Troccoli A., Duncan L., Subbotin S., Waeyenberge L., Coyne L., Brentu F., Inserra R. (2012). Pratylenchus speijeri n. sp. (Nematoda: Pratylenchidae), a new root-lesion nematode pest of plantain in West Africa. Journal of Nematology.

[j_jofnem-2022-0057_ref_011] Glazer I., Orion D. (1983). Estudios sobre anhidrobiosis de Pratylenchus thornei. Journal of Nematology.

[j_jofnem-2022-0057_ref_012] Guzmán O., Castaño J. (2004). Reconocimiento de nematodos fitopatógenos en plátanos dominico hartón (Musa AAB Simmonds), África, FHIA-20 y FHIA-21, en la granja Montelindo, municipio de Palestina (Caldas), Colombia. Revista de la Academia Colombiana de Ciencias Exactas Físicas y Naturales.

[j_jofnem-2022-0057_ref_013] Guzmán O., Castaño J. (2007). Manejo de nematodos en plátano (Musa spp.) mediante tratamiento de la semilla. Fitopatologia Colombiana.

[j_jofnem-2022-0057_ref_014] Hall T. A. (1999). BioEdit: A user-friendly biological sequence alignment editor and analysis program for windows 95/98/NT. Nucleic Acids Symposium Series.

[j_jofnem-2022-0057_ref_015] Handoo Z. A., Yan G., Kantor M. R., Huang D., Chowdhury I. A., Plaisance A., Bauchan G. R., Mowery J. D. (2021). Morphological and molecular characterization of Pratylenchus dakotaensis n. sp. (Nematoda: Pratylenchidae), a new root-lesion nematode species on soybean in North Dakota, USA. Plants.

[j_jofnem-2022-0057_ref_016] Huang D., Yan G. (2017). Specific detection of the root-lesion nematode Pratylenchus scribneri using conventional and real-time PCR. Plant Disease.

[j_jofnem-2022-0057_ref_017] Inserra R., Kaplan D., Maia Dos Santos J., Duncan L., Vovlas N., Dunn D., Troccoli A. (2001). Pratylenchus jaehni sp. n. from citrus in Brazil and its relationship with P. coffeae and P. loosi (Nematoda: Pratylenchidae) Nematology.

[j_jofnem-2022-0057_ref_018] Keane T., Creevey C., Pentony M., Naughton T., McInerney J. (2006). Assessment of methods for amino acid matrix selection and their use on empirical data shows that ad hoc assumptions for choice of matrix are not justified. BMC Evolutionary Biology.

[j_jofnem-2022-0057_ref_019] Kim D., Chun J. Y., Lee K. Y. (2016). Morphological and molecular identification of two root-lesion nematodes, Pratylenchus kumamotoensis and P. pseudocoffeae in Korea. ZooKeys.

[j_jofnem-2022-0057_ref_020] Kolombia Y., Ogundero O., Olajide M., Viaene N., Kumar L., Coyne D., Bert W. (2020). Morphological and molecular characterization of Pratylenchus species from Yam (Dioscorea spp.) in West Africa. Journal of Nematology.

[j_jofnem-2022-0057_ref_021] Li Y., Lu Q., Wang S., Liu Y., Wang K., Yuan H., Li H. (2018). Discovery of a root-lesion nematode, Pratylenchus scribneri, infecting corn in Inner Mongolia, China. Plant Disease.

[j_jofnem-2022-0057_ref_022] Li Y., Xia Y., Liu Y., Hao P., Sun B., Li H., Wang K. (2020). Discovery of root-lesion nematode, Pratylenchus coffeae, infesting sesame in China. Plant Disease.

[j_jofnem-2022-0057_ref_023] Liu X., Wang H., Lin B., Tao Y., Zhuo K., Liao J. (2016). Loop-mediated isothermal amplification based on the mitochondrial COI region to detect Pratylenchus zeae. European Journal of Plant Pathology.

[j_jofnem-2022-0057_ref_024] Luambano N., Kashandoa B., Masungaa M., Mwenisongolea A., Mziraya M., Mbagaa J., Polinia R., Mgonjab D. (2019). Status of Pratylenchus coffeae in banana-growing areas of Tanzania. Physiological and Molecular Plant Pathology.

[j_jofnem-2022-0057_ref_025] Mirghasemi S. N., Fanelli E., Troccoli A., Jamali S., Sohani M., De Luca F. (2019). Molecular variability of the root-lesion nematode, Pratylenchus loosi (Nematoda: Pratylenchidae), from tea in Iran. European Journal of Plant Pathology.

[j_jofnem-2022-0057_ref_026] Moens T., Araya M., Swennen R., De Waele D. (2006). Reproduction and pathogenicity of Helicotylenchus multicinctus Meloidogyne incognita and Pratylenchus coffeae, and their interaction with Radopholus similis on Musa. Nematology.

[j_jofnem-2022-0057_ref_027] Múnera G. E. (2008). Biodiversity of phytoparasitic nematodes associated with Musaceae and fruit crops in Colombia. Ph. D. Thesis.

[j_jofnem-2022-0057_ref_028] Múnera G., Bert W., Decraemer W. (2009). Morphological and molecular characterisation of Pratylenchus araucensis n. sp. (Pratylenchidae), a root-lesion nematode associated with Musa plants in Colombia. Nematology.

[j_jofnem-2022-0057_ref_029] Mwamula A. O., Kabir M. F., Lee G., Choi I. H., Kim Y. H., Bae E. J., Lee D. W. (2020). Morphological characterisation and molecular phylogeny of several species of Criconematina (Nematoda: Tylenchida) associated with turfgrass in Korea, as inferred from ribosomal and mitochondrial DNA. Nematology.

[j_jofnem-2022-0057_ref_030] Nguyen H. T., Trinh Q. P., Couvreur M., Singh P. R., Decraemer W., Bert W. (2018). Molecular and morphological characterisation of a new root-lesion nematode, Pratylenchus horti n. sp. (Tylenchomorpha Pratylenchidae), from Ghent University Botanical Garden. Nematology.

[j_jofnem-2022-0057_ref_031] Orión D. (2000). Nematodes of agricultural importance in Israel. Nematology.

[j_jofnem-2022-0057_ref_032] Orión D., Amir J., Krikun J. (1984). Observaciones de campo sobre Pratylenchus thornei y sus efectos sobre el trigo en condiciones áridas. Revue Nématologie.

[j_jofnem-2022-0057_ref_033] Orión D., Krikun J., Sullami M. (1979). La distribución, patogenicidad y ecología de Pratylenchus thornei en el norte del Negev. Phytoparasitica.

[j_jofnem-2022-0057_ref_034] Qing X., Bert W., Gamliel A., Bucki P., Duvrinin S., Alon T., Braun S. (2019). Phylogeography and molecular species delimitation of Pratylenchus capsici n. sp., a new root – Lesion nematode in Israel on pepper (Capsicum annuum). Phytopathology.

[j_jofnem-2022-0057_ref_035] Ravichandra N., Ravichandra N. G. (2014). Horticultural nematology.

[j_jofnem-2022-0057_ref_036] Riascos-Ortiz D., Mosquera-Espinosa A. T., De Agudelo F. V., de Oliveira C., Muñoz-Flórez J. E. (2019). Morpho-molecular characterization of Colombian and Brazilian populations of Rotylenchulus associated with Musa spp. Journal of Nematology.

[j_jofnem-2022-0057_ref_037] Riascos-Ortiz D., Mosquera-Espinosa A., Varón de Agudelo F., Muñoz-Florez J. (2021). Importancia relativa de nematodos fitoparásitos asociados a Musa spp. y las interrelaciones entre los géneros de mayor valor de prominencia. Fitopatología Colombiana.

[j_jofnem-2022-0057_ref_038] Riascos-Ortiz D., Mosquera-Espinosa A. T., Varón de Agudelo F., Oliveira C. M. G., Muñoz Flórez J. E., Chaudhary K. K., Meghvansi M. K. (2022). Sustainable management of nematodes in agriculture, vol. 1: Organic management. Sustainability in Plant and Crop Protection, vol. 18.

[j_jofnem-2022-0057_ref_039] Rosa J. M. O., Oliveira A. de S., Alexandre L. J., Amauri S., Oliveira C. M. G. (2014). Nematoides fitoparasitas associados à mandioca na Amazônia brasileira. Acta Amazonica.

[j_jofnem-2022-0057_ref_040] Sasser J., Freckman D., Veech J., Dickson D. (1987). Vistas in nematology.

[j_jofnem-2022-0057_ref_041] Shokoohi E., Abolafia J., Mashela P. W., Divsalar N. (2018). New data on known species of Hirschmanniella and Pratylenchus (Rhabditida, Pratylenchidae) from Iran and South Africa. Journal of Nematology.

[j_jofnem-2022-0057_ref_042] Singh P., Nyiragatare A., Janssen T., Couvreur M., Decraemer W., Bert W. (2018). Morphological and molecular characterisation of Pratylenchus rwandae n. sp. (Tylenchida: Pratylenchidae) associated with maize in Rwanda Nematology.

[j_jofnem-2022-0057_ref_043] Souza R. (2008). Plant-parasitic nematodes of coffee: Springer, Campos dos Goytacazes.

[j_jofnem-2022-0057_ref_044] Talavera M., Valor H., Tobar A. (1998). Viabilidad post-anhidrobiótica de Pratylenchus thornei y Merlinius brevidens. Phytoparasitica.

[j_jofnem-2022-0057_ref_045] Tamura K., Stecher G., Peterson D., Filipski A., Kumar S. (2013). MEGA6: Molecular evolutionary genetics analysis version 6.0. Molecular Biology Evolution.

[j_jofnem-2022-0057_ref_046] Troccoli A., Subbotin S., Chitambar J., Janssen T., Waeyenberge L., Stanley J., Duncan L., Agudelo P., Múnera Uribe G., Franco J., Inserra R. (2016). Characterisation of amphimictic and parthenogenetic populations of Pratylenchus bolivianus Corbett, 1983 (Nematoda: Pratylenchidae) and their phylogenetic relationships with closely related species. Nematology.

[j_jofnem-2022-0057_ref_047] Varón de Agudelo F., Castillo G. P. (2001). Seminario taller sobre identificación de nematodos de importancia en agricultura. Guía Práctica. ASCOLFI, Palmira, marzo 28-30 de 2001.

[j_jofnem-2022-0057_ref_048] Wang H., Zhuo K., Liao J. (2016). Morphological and molecular characterization of Pratylenchus hippeastri, A new record of root-lesion nematode associated with apple in China. Pakistan Journal of Zoology.

[j_jofnem-2022-0057_ref_049] Wang H., Zhuo K., Ye W., Liao J. (2015). Morphological and molecular characterisation of Pratylenchus parazeae n. sp. (Nematoda: Pratylenchidae) parasitizing sugarcane in China. European Journal of Plant Pathology.

[j_jofnem-2022-0057_ref_050] Wujian C., Yong B., Rong Wu., Xiaojia L., Mingzhe Z., Zhiyi W., Yang W., Xuyao W., Xi C. (2014). Root-lesion nematode Pratylenchus hippeastri, on Acer plmatum imported from Japan. Acta Agriculturae Zhejiangensis.

[j_jofnem-2022-0057_ref_051] Zamora T. Z., Padilla W. P., Archidona-Yuste A., Cantalapiedra-Navarrete C., Liebanas G., Palomares-Rius J. E., Castillo P. (2016). Root-lesion nematodes of the genus Pratylenchus (Nematoda: Pratylenchidae) from Costa Rica with molecular identification of P. gutierrezi and P. panamaensis topotypes. European Journal of Plant Pathology.

[j_jofnem-2022-0057_ref_052] Zimmermann A. W. P. (1898). De nematoden der koffiewortels. Deel I. Mededelingen uit’s Lands Plantentuin.

[j_jofnem-2022-0057_ref_053] Zuñiga G., Ortiz R., Varón De Agudelo F. (1979). Nematodos asociados con el cultivo del plátano (Musa AAB ABB) en el Valle del Cauca. Fitopatologia Colombiana.

